# HIF-1α-mediated augmentation of miRNA-18b-5p facilitates proliferation and metastasis in osteosarcoma through attenuation PHF2

**DOI:** 10.1038/s41598-022-13660-w

**Published:** 2022-06-21

**Authors:** Peng Luo, Yan-dong Zhang, Feng He, Chang-jun Tong, Kai Liu, He Liu, Shi-zhuang Zhu, Jian-zhou Luo, Bing Yuan

**Affiliations:** 1 Department of Orthopedics, Huazhong University of Science and Technology Union Shenzhen Hospital, Shenzhen, 518000 China; 2 Guangdong Medical University, Zhanjiang, 524023 China; 3 Department of Orthopedic Surgery, The Third Hospital of Hebei Medical University, Shijiazhuang, 050051 China; 4 Department of Orthopedics, The Fifth Hospital of Wuhan/The Second Affiliated Hospital of Jianghan University, Wuhan, 430050 China

**Keywords:** Cancer, Biomarkers, Oncology

## Abstract

Extensive evidence has explored the involvement of microRNAs (miRNAs) in osteosarcoma (OS). Limitedly, the concrete function of microRNA-18b-5p (miR-18b-5p) in OS remains unexplored and largely elusive. Here, we validated that miR-18b-5p significantly elevated in OS via analyzing the data from GEO database. The results showed that miR-18b-5p was overexpressed in human OS tissues and cell lines. The clinical evidence suggested that high level of miR-18b-5p was negatively correlated with the poor prognosis of OS. Meanwhile, miR-18b-5p upregulation facilitated the proliferation and metastasis of OS cells in vitro and in vivo. The mechanism exploration demonstrated that miR-18b-5p acted as a potential inhibitor of PHF2, a tumor suppressor gene, at post-transcriptional level. Moreover, hypoxia induced gene expression of miR-18b-5p was clarified to be transcriptionally mediated by HIF-1α. The clinicopathological analysis in samples of OS patients further supported that miR-18b-5p had a positive correlation with HIF-1α expression, and negative correlation with PHF2. Collectively, the present study uncovered a new molecular mechanism of OS tumorigenesis and development and miR-18b-5p might be a prognostic biomarker and potential therapeutic target for OS treatment.

## Introduction

Osteosarcoma (OS) is the most common primary bone malignant tumor in adolescents and elderly people over 65^1^. Its poor prognosis is mainly due to rapid growth and early metastasis. Approximately 20% of patients have developed distant invasive metastases at the time of consultation. Ultimately, they lost the opportunity for radical surgical treatment^2^. Although great efforts and progress have been made to ensure the best therapeutic effect, the 5-year survival rate of patients with metastatic OS is less than 30%^3,4^. Therefore, it is necessary to explore new molecular mechanisms from a new perspective to provide new targets and strategies for the diagnosis and treatment of OS.

Oxygen concentration in solid tumor tissue is generally less than 2% due to rapid growth, insufficient local blood vessels to provide adequate blood supply, destruction of microcirculation, anemia and other reasons^5^. OS, as a form of solid tumor, should also be hypoxic in the tumor tissue^6^. Hypoxic tumor microenvironment promotes tumor progression, metastasis and the formation of drug resistance, which is related to the occurrence, development, therapeutic effect and prognosis of various tumors^7^. Hypoxia inducible factor-1 (HIF-1) complex, composed of HIF-1α and HIF-1β subunits, plays a key role in mediating hypoxia reactions^8^. It has been reported that HIF-1α, which is enhanced in stability, mediates adaptive survival of tumor cells by regulating gene expression by binding to hypoxia-responsive elements (HREs, 5′ -RCGTG-3′) in target gene enhancer or promoter regions under hypoxia^9^. Meanwhile, a literature has been demonstrated that HIF-1α increased the incidence of OS via inhibiting the protein expression of IDH-1^10^. However, the exact mechanism by which HIF-1α is involved in OS under hypoxic microenvironment remains equivocal. Therefore, there is a need to identify additional driver mechanisms of the occurrence, progression, invasion and metastasis of OS cells under hypoxia, so as to provide new ideas and drug targets for the clinical diagnosis and treatment of tumors and effectively improve the quality of life of patients.

PHF2 gene, encoding plant homeodomain (PHD) and Jumonji C (JmjC) domain-containing proteins, is identified as transcriptional regulators affecting gene expression by demethylation of histone lysine^11–13^. Research has clarified that PHF2 was downregulated in squamous cell carcinoma, Gastric and Colorectal Cancers and other malignant tumors^14–16^. Moreover, the deletion/methylation of PHF2 gene leads to downregulation of PHF2 expression in breast cancer^17^. Meanwhile, low expression of PHF2 is related to the aggressiveness and poor prognosis of clear cell renal cell carcinoma (CCRCC)^18^. The overexpression of PHF2 in esophageal squamous cell carcinoma (ESCC) is correlated to decreased overall survival rate of ESCC patients^19^. Mechanism studies have elucidated that PHF2 positively regulated p53 expression via demethylation of histone H3K9 methylation at p53 promoters, indicating that PHF2 acted as a tumor suppressor gene and played a negative regulatory role in tumor genesis and development^16^. Whereas, the function and underlying molecular mechanisms of PHF2 in OS cells are rarely discussed.

MicroRNAs (miRNAs) are a class of endogenous regulatory small non-coding RNA, which identify complementary sequences of its target genes in the 3′ -untranslated region (UTRs), leading to mRNA degradation, translation inhibition or activation^20,21^. miRNA plays a role in regulating cell proliferation, differentiation, apoptosis and ontogenesis^22^. Various studies have elucidated that miRNA is closely related to the occurrence and development of OS^23,24^. Here, we identified miR-18b-5p significantly elevated in OS via analyzing the data from GEO database. Our results suggest that upregulation of miR-18b-5p in OS plays a crucial role in the proliferation and metastasis of OS cells. Additionally, we make sense out of the regulative mechanism of miR-18b-5p in OS. Our results further broaden our understanding of the action and mechanism of miRNAs in OS, and can potentially provide new avenues for OS treatment.

## Results

### miR-18b-5p is upregulated in OS and associated with poor clinical outcome

**Figure 1:**
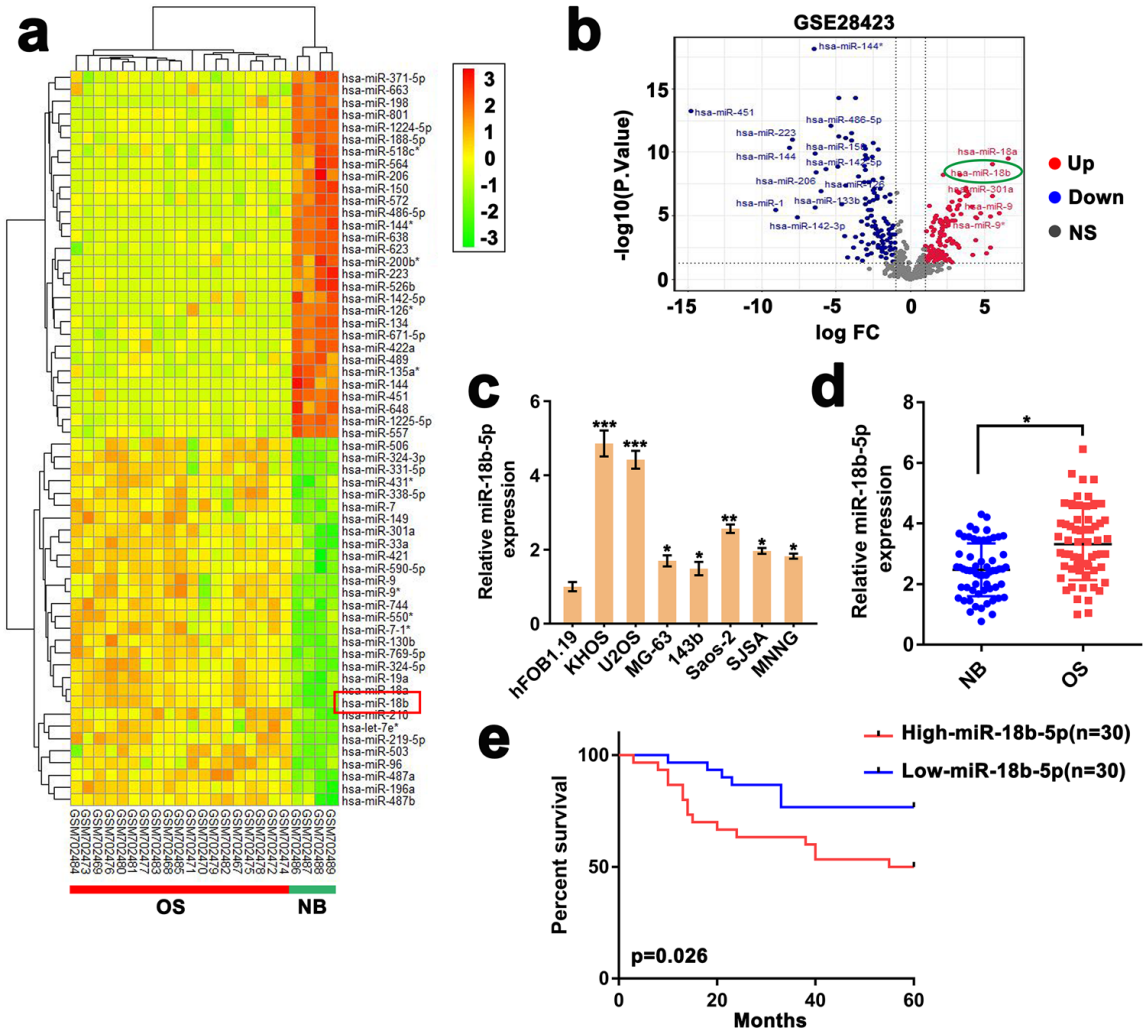
miR-18b-5p is upregulated in OS and associated with poor clinical outcome. (**a**) Heatmap of microarray data obtained from GEO datasets (GSE28423) showed 60 most upregulated and downregulated miRNAs in OS cells and normal osteocytes. The image was created by RStudio (pheatmap). (**b**) The volcano plot showed differential expressed miRNAs (log2 fold change > 2, *p* < 0.001). The image was generated by RStudio (ggplot2). (**c**) RT-qPCR assay showed the miR-18b-5p expression in 7 OS cell lines compared with normal bone cells (hFOB1.19). (**d**) RT-qPCR assay showed the miR-18b-5p expression in 60 OS tissues and paired adjacent normal tissues (NB). (**e**) Kaplan–Meier curves analysis displayed the percentage survival of 60 OS patients in high and low miR-18b-5p groups. The 50th percentile of miR-18b-5p expression was a cutoff point divided into “high” and “low”.

To identify miRNAs involved in OS progression, we analyzed the expression of differential expressed miRNAs in 19 OS cell lines and 4 normal bones extracted from GEO Datasets (GSE28423). Through bioinformatics analysis^25,26^, we observed that 80 miRNAs were upregulated (log2 fold change > 2, *p* < 0.05) and 75 were down-regulated (log2 fold change < -2, *p* < 0.05) (Fig. 1a). Among miRNAs with a significant difference (log2 fold change > 2, *p* < 0.001), miR-18a, miR-301a and miR-9 had been fully identified as a tumor-relating miRNA in OS in previous studies^27–29^. Therefore, we selected miR-18b-5p for further investigation (Fig. 1b). To validate the microarray results, we performed RT-qPCR to quantify miR-18b-5p in both OS cells (MG-63, 143b, KHOS, U2OS, Saos-2, SJSA and MNNG) and osteocytes (hFOB1.19). As demonstrated, miR-18b-5p was significantly upregulated in OS cells compared with normal osteocytes (Fig. 1c). Additionally, miR-18b-5p level was assessed in 60 OS tissues and the paired adjacent normal tissues by RT-qPCR. The results suggested that miR-18b-5p was remarkably upregulated in OS tissues compared with that in normal tissues (Fig. 1d). Furthermore, analysis of overall survival showed that the patients with high level of miR-18b-5p showed shorter overall survival rate (Fig. 1e). Collectively, these results indicated that miR-18b-5p was highly expressed in OS and related to poor prognosis of OS patients.

### Upregulation of miR-18b-5p accelerates development of OS

**Figure 2:**
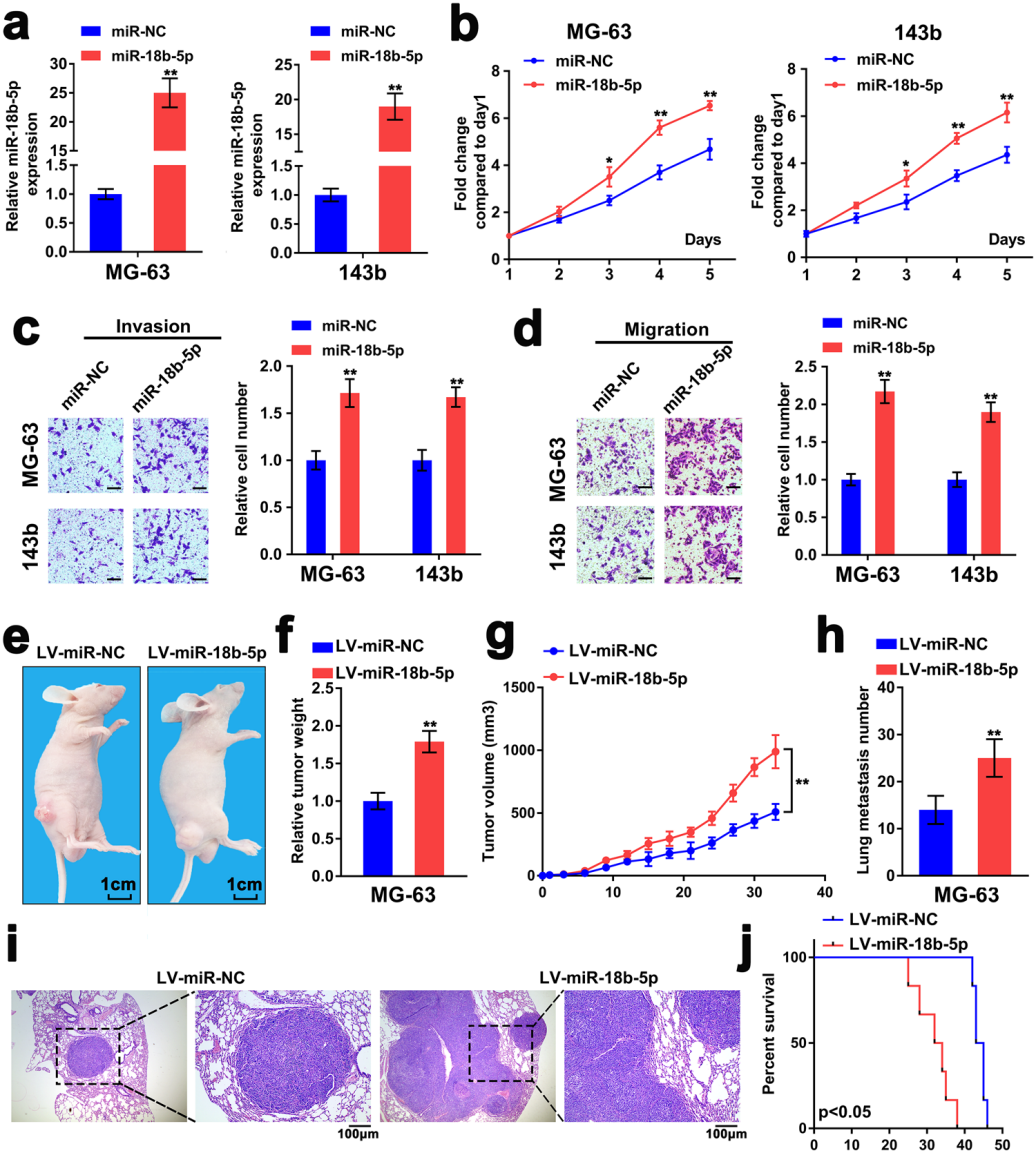
Upregulation of miR-18b-5p accelerates development of OS. (**a**) RT-qPCR was performed to detect miR-18b-5p expression in MG-63/143b cells transfected with miR-18b-5p mimic or miR-NC. (**b**) The cell viability of MG-63/143b cells was evaluated by the MTT method. (**c**,**d**) The capacity of invasion and migration of MG-63/143b cells was valuated by the transwell assay. Scale bar = 100 μm. (**e**) Xenograft tumor of nude mice subcutaneously injected with MG-63 with stably miR-18b-5p overexpression. (**f**,**g**) The weight and volume of xenograft tumor of nude mice. (**h**–**j**) The nude mice was injected with MG-63 with stably miR-18b-5p overexpression from tail vein. The number of lung metastasis of nude mice counted via H&E staining (**h**). The images of H&E staining of lung metastasis (**i**). The survival of nude mice was analyzed (**j**).

To identify the biological function of miR-18b-5p in OS, the effects of upregulated and downregulated miR-18b-5p on cell proliferation and motility were investigated. Compared with other OS cells, miR-18b-5p expression was most elevated in KHOS and U2OS cells and lowest in MG-63 and 143b cells, so we inhibited the expression of miR-18b-5p in KHOS and U2OS cells and up-regulated the expression of miR-18b-5p in MG-63 and 143b cells. MiRNA mimic and inhibitor were applied to elevate or decrease endogenous miR-18b-5p, respectively (Fig. 2a, Fig. S1A). MTT assay indicated significant enhanced proliferation of OS cells with miR-18b-5p overexpression compared with the control group, whereas the opposite effects were found following miR-18b-5p depletion (Fig. 2b, Fig. S1B). In addition, the results of transwell assay suggested that miR-18b-5p overexpression accelerated the invasion and migration of OS cells (Fig. 2c,d). In contrast, miR-18b-5p inhibition repressed cell invasion and migration (Fig. S1C, D).

To validate the biological function of miR-18b-5p in vivo, MG-63 cells, transfected with recombinant lentiviral vector LV-miR-18b-5p (LV-miR-NC as control), were utilized to establish xenograft and metastasis models by subcutaneous or tail vein injection. The results showed that compared with LV-miR-NC group, miR-18b-5p overexpression significantly increased the tumor growth (Fig. 2e–g). Consistent with the results of the in vitro assay, miR-18b-5p overexpression enhanced the metastasis of OS cells in vivo (Fig. 2h,i). Meanwhile, miR-18b-5p upregulation group exhibited shorter survival rate compared with the control group (Fig. 2j). Thus, these results revealed that miR-18b-5p functioned as an oncogenic miRNA in OS.

### miR-18b-5p modulates the expression of PHF2 through post-transcriptional regulation

**Figure 3:**
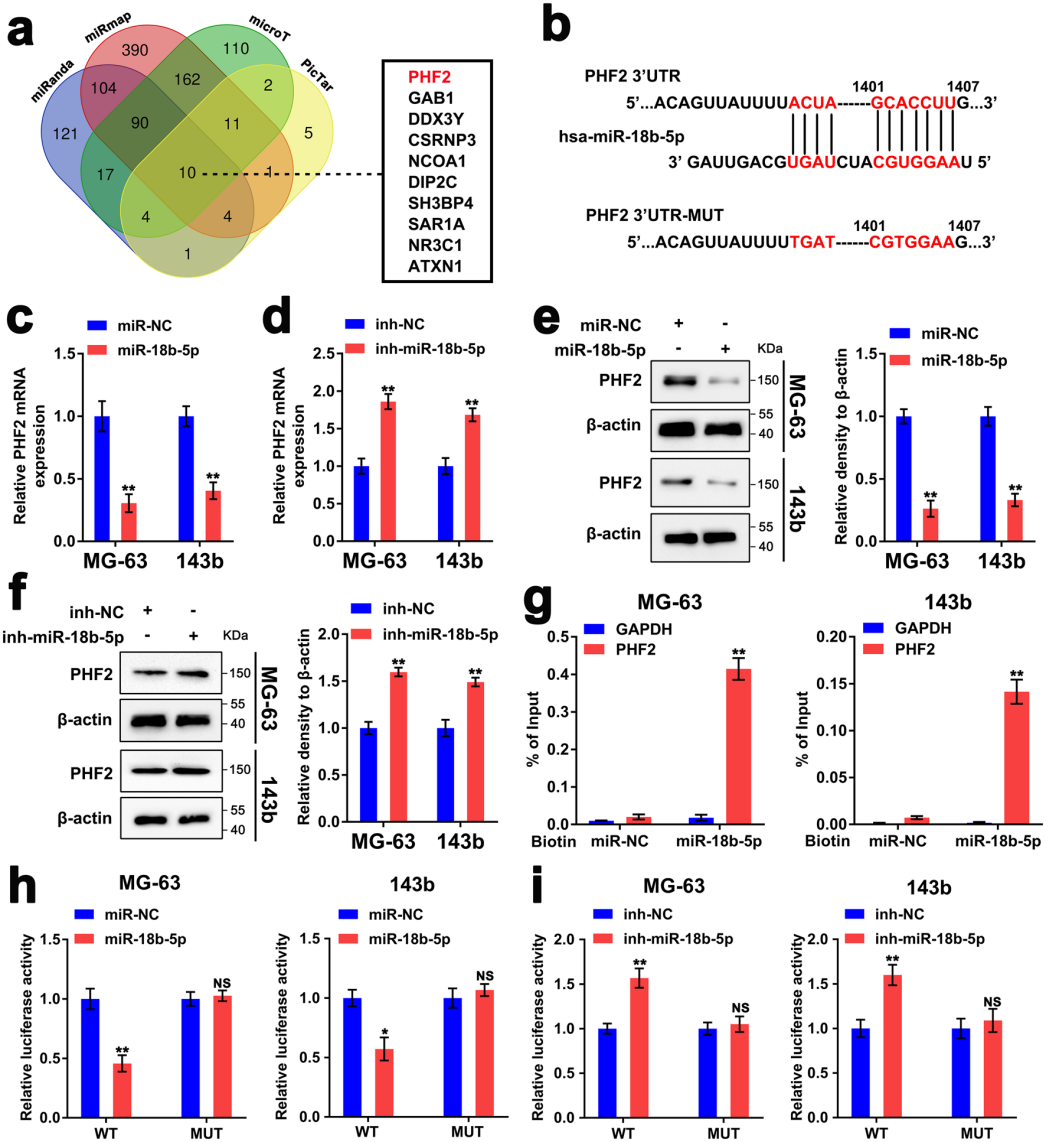
miR-18b-5p modulates the expression of PHF2 through post-transcriptional regulation. (**a**) Venn diagram showed the predicted targets of miR-18b-5p from databases (miRanda, miRmap, microT and PicTar). The image was created using Online tools of Bioinformatics Evolutionary Genomics (http://bioinformatics.psb.ugent.be/webtools/Venn/). (**b**) Schematic diagram showed the predicted interaction between 3′-UTR-PHF2- mRNA and miR-18b-5p. The mutated sequences of the 3′-UTR-PHF2- mRNA was displayed. (**c**,**d**) RT-qPCR assay showed the expression of PHF2 mRNA in MG-63/143b cells with miR-18b-5p overexpression or inhibition. (**e**,**f**) Western blotting assay showed the protein level of PHF2 in miR-18b-5p upregulated or downregulated MG-63 and 143b cells. (**g**) Biotin-based pulldown assay detected the enrichment of PHF2 on biotinylated miR-NC and miR-18b-5p probe with the mRNA of GAPDH acted as control. (**h**,**i**) The luciferase reporter assay accessed luciferase activity of miR-18b-5p upregulated or downregulated MG-63 and 143b cells transfected with luciferase reporter plasmid carrying WT-3′-UTR-PHF2 or MUT-3′-UTR-PHF2.

According to the reported methodology^30,31^, the bioinformatics databases (miRanda, miRmap, microT and PicTar) were applied to predict potential target genes of miR-18b-5p and explore underlying mechanisms involved. The results of Venn’s diagram showed that there were 10 genes, including PHF2, which might be downstream targets of miR-18b-5p (Fig. 3a,b). Since it has been reported that the histone demethylase PHF2 is considered a putative anti-tumor gene, and is associated with p53 as a tumor suppressor in cancer^16^. Thus, we wondered whether miR-18b-5p exerted its function through targeting PHF2. The RT-qPCR results indicated that overexpression of miR-18b-5p decreased the mRNA level of PHF2, while inhibition of miR-18b-5p increased the mRNA level of PHF2 (Fig. 3c,d). Additionally, overexpressed miR-18b-5p dramatically decreased PHF2 protein levels, while transfection of miR-18b-5p inhibitors promoted PHF2 protein level in both MG-63 and 143b cells (Fig. 3e,f). These results indicated that miR-18b-5p negatively regulated expression of PHF2 at the post-transcriptional level. In order to validate that miR-18b-5p specifically bound to the 3′UTR of PHF2 mRNA, immuno-precipitation using biotin labeled miR-18b-5p was conducted with biotin-miR-NC served as a negative control. The result showed that PHF2 mRNA was only detected in the biotin labeled miR-18b-5p group (Fig. 3g). Subsequently, we performed the luciferase reporter assay to further prove the direct interaction between miR-18b-5p and PHF2 mRNA. We performed luciferase assays by transfecting MG-63 and 143b cells with luciferase reporters carrying Wild or Mutant type (WT, MUT) of PHF2-mRNA-3′ UTR sequences along with miR-18b-5p overexpression or depletion. The results suggested that miR-18b-5p overexpression significantly decreased luciferase activity of WT-3′ UTR-PHF2 cells, while miR-18b-5p inhibition significantly increased luciferase activity of WT-3′ UTR-PHF2 cells (Fig. 3h,i). Neither miR-18b-5p overexpression nor miR-18b-5p inhibition affect luciferase activity of MUT-3′ UTR-PHF2 cells. Therefore, these results indicated that PHF2 was a downstream target of miR-18b-5p.

### PHF2 is responsible for miR-18b-5p-mediated cell proliferation, invasion and migration

**Figure 4:**
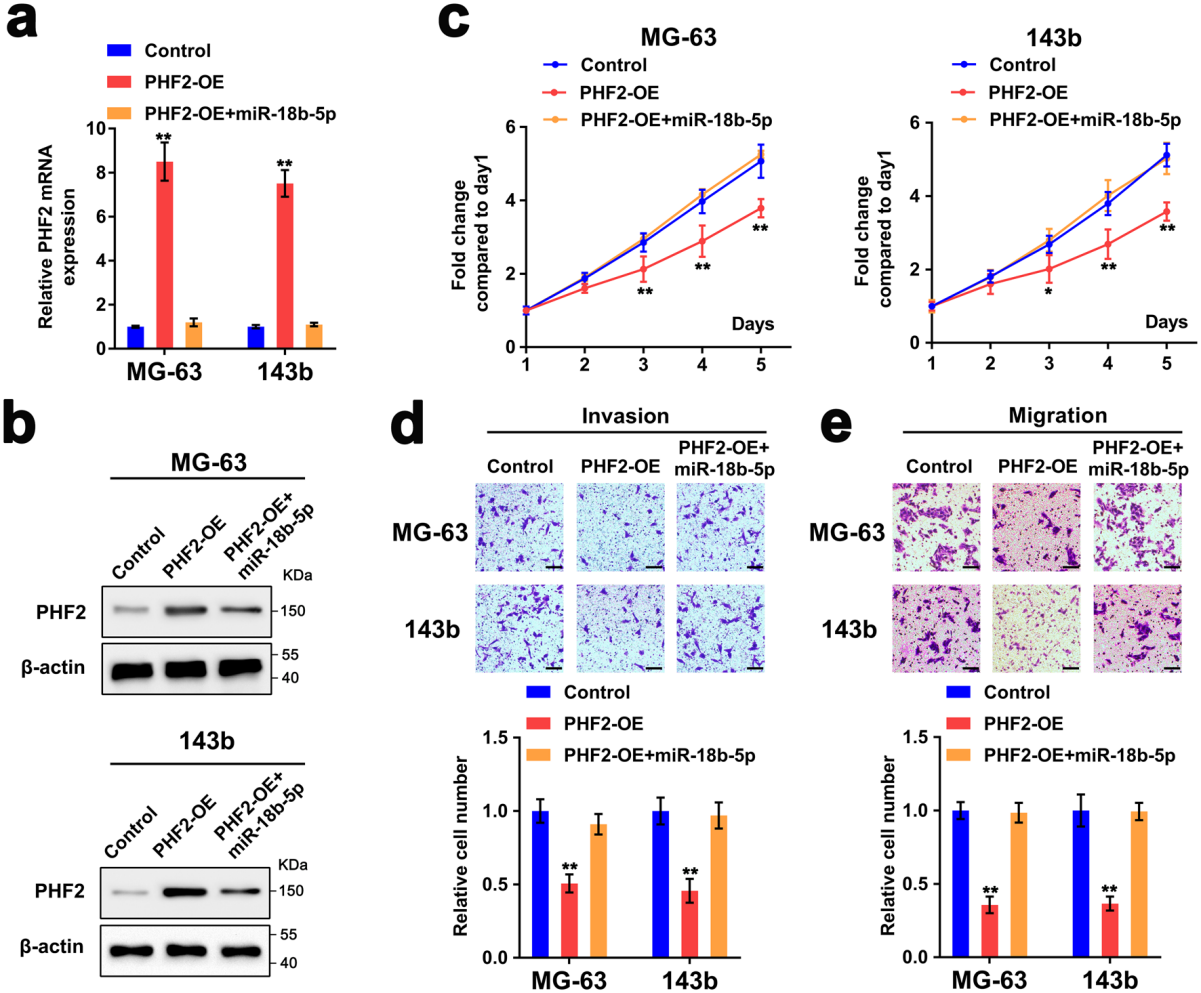
PHF2 is responsible for miR-18b-5p-mediated cell proliferation, invasion and migration. (**a**,**b**) RT-qPCR and western blotting results showed the mRNA and protein expression of PHF2 in MG-63/143b cells transfected with PHF2 overexpression plasmid, miR-18b-5p mimic or negative control. (**c**–**e**) The proliferation, invasion and migration of MG-63/143b cells, transfected with PHF2 overexpression plasmid, miR-18b-5p mimic or negative control, was evaluated by the MTT and transwell assays. Scale bar = 100 μm.

To identify the ability of miR-18b-5p to promote tumor progression through inhibiting PHF2, we co-transfected MG-63 and 143b cells overexpressing PHF2 or scrambled vector with miR-18b-5p mimic or miR-NC (Fig. 4a,b). The results of MTT assay indicated that PHF2 overexpression significantly decreased the cell proliferation, which could be rescued by miR-18b-5p upregulation (Fig. 4c). Moreover, transwell assay showed that overexpression of miR-18b-5p rescued the suppressive effects of PHF2 overexpression on OS invasion and migration (Fig. 4d,e). Collectively, these results revealed that miR-18b-5p promoted proliferation, invasion and migration via targeting PHF2.

### Hypoxia-driven HIF-1α is critical for upregulation of miR-18b-5p in OS

**Figure 5:**
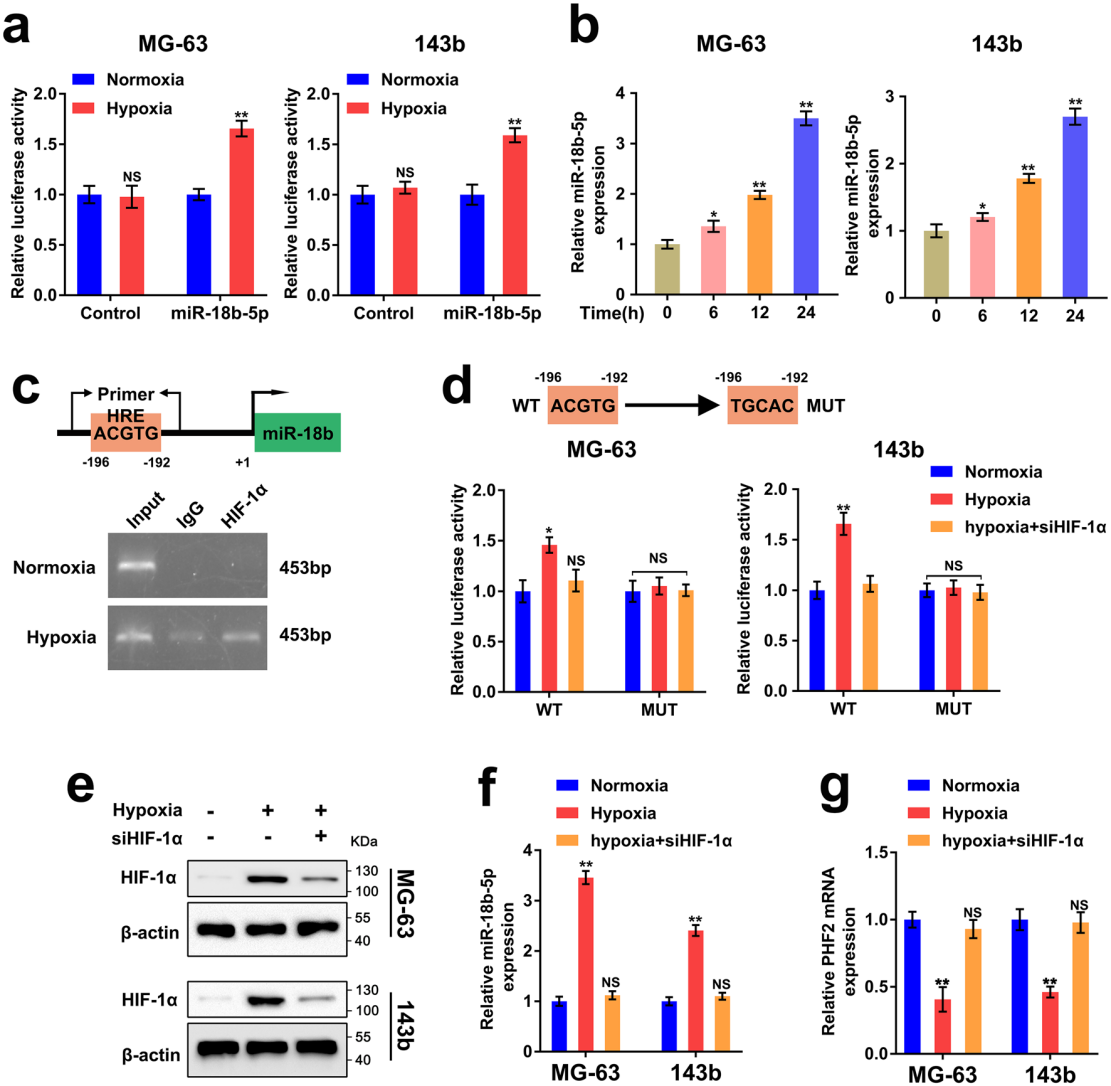
Hypoxia-driven HIF-1α is critical for upregulation of miR-18b-5p in OS. (**a**) The luciferase reporter assay accessed luciferase activity of MG-63/143b cells transfected with luciferase reporter plasmid carrying miR-18b promoter sequence or scrambled vector under normoxia and hypoxia. (**b**) RT-qPCR assay showed miR-18b-5p expression in MG-63/143b cells cultured under hypoxia for 0 h, 6 h, 12 h and 24 h. (**c**) (Up) Schematic diagram showed the predicted HRE site on the promoter of miR-18b. (Down) ChIP assay using antibodies against HIF-1α or IgG were performed to validate the binding of HIF-1α on the promoter of miR-18b. (**d**) The luciferase reporter assay accessed luciferase activity of MG-63 and 143b cells transfected with luciferase reporter plasmid carrying WT- miR-18b or MUT- miR-18b, siRNA targeting HIF-1α and negative control under normoxia or hypoxia. (**e**) Western blotting assay accessed protein level of HIF-1α in MG-63/143b cells transfected with siRNA targeting HIF-1α and negative control under normoxia or hypoxia. (**f,g**) RT-qPCR assay showed the expression of miR-18b-5p and PHF2 mRNA in MG-63/143b cells transfected with siRNA targeting HIF-1α and negative control under normoxia or hypoxia.

Since researchers had elucidated that hypoxic microenvironment played a vital role in mediating multiple processes of the malignant progression of OS^32,33^. Therefore, we ought to validate whether hypoxia could induce upregulation of miR-18b-5p in OS. To validate, we designed luciferase reporters containing the promoter sequence of miR-18b-5p host gene, miR-18b, or negative control sequence. The result indicated that the activity of miR-18b promoter was remarkably elevated under hypoxia (Fig. 5a). Meanwhile, RT-qPCR assay indicated that the expression of miR-18b-5p was remarkably increased with the extension of hypoxic culture time (Fig. 5b, Fig. S2A). Next, we found a HRE element in the miR-18b promoter via analyzing the promoter sequence of miR-18b promoter. Chromatin immunoprecipitation (ChIP) assay suggested that HIF-1α could bind to the promoter of miR-18b under hypoxia (Fig. 5c). In order to further demonstrate that HIF-1α regulates miR-18b-5p by binding to the HRE site of miR-18b promoter, the MG-63 and 143b cells were transfected with WT or MUT plasmid carrying promoter sequence of miR-18b. The luciferase assay revealed that hypoxia significantly enhanced the activity of miR-18b promoter in WT cells, which was reversed by HIF-1α silencing (Fig. 5d,e). Additionally, RT-qPCR results indicated that HIF-1α inhibition reversed the miR-18b-5p expression induced by hypoxia (Fig. 5f). Consistently, the expression of PHF2 was remarkably decreased under hypoxia, which was restored by HIF-1α inhibition (Fig. 5g). Therefore, the results above revealed that hypoxia induced HIF-1α is responsible for the upregulation of miR-18b-5p in OS under hypoxic microenvironment.

### Hypoxia induced miR-18b-5p promotes OS via miR-18b-5p–PHF2 axis

**Figure 6:**
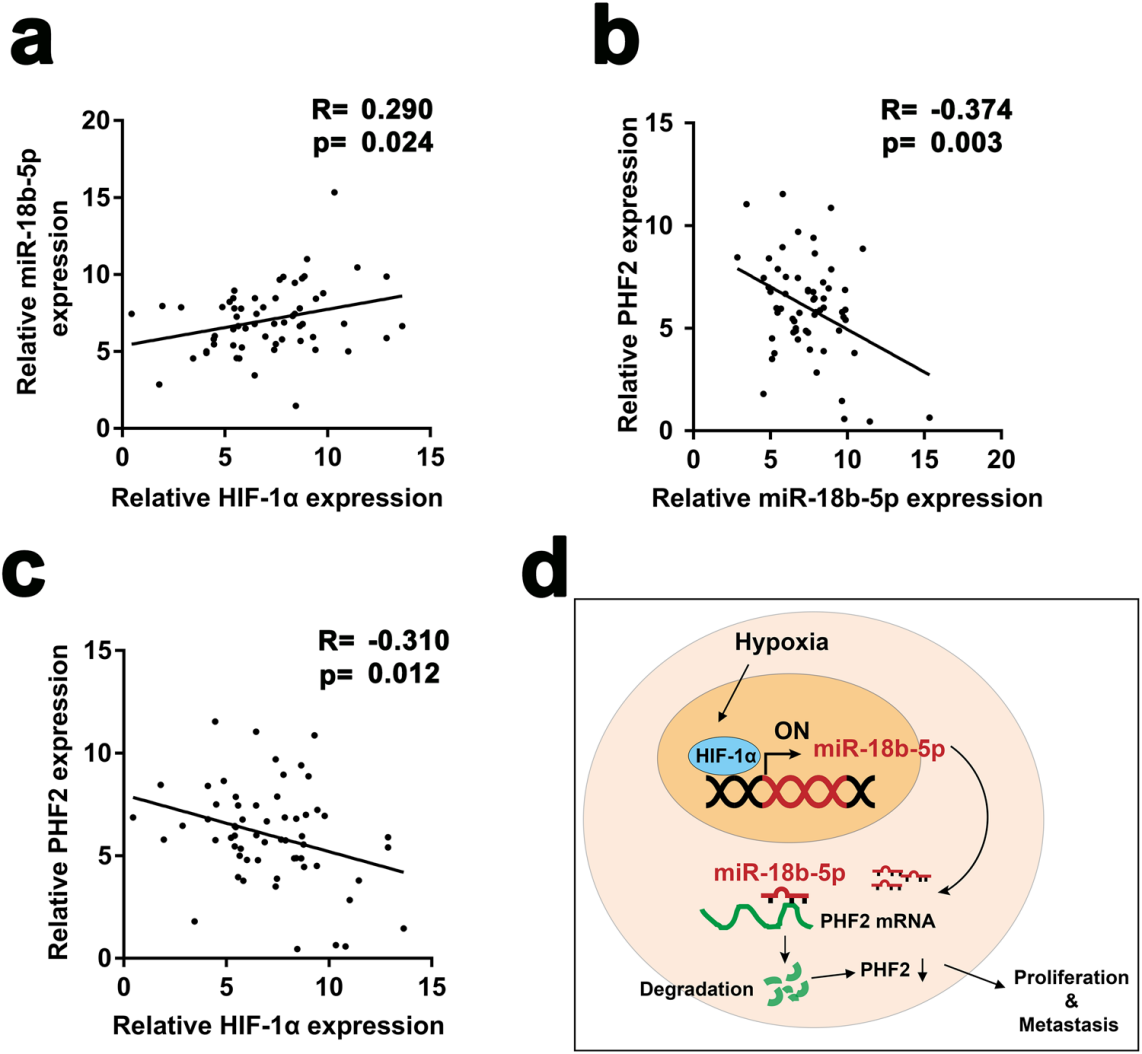
Hypoxia induced miR-18b-5p promotes OS via HIF-1α-miR-18b-5p-PHF2 axis. (**a**–**c**) Pearson correlation analysis showed the correlation between miR-18b-5p and HIF-1α, miR-18b-5p and PHF2, PHF2 and HIF-1α in OS tissues determined by RT-qPCR. (**d**) Schematic diagram showed the molecular mechanism of miR-18b-5p involved in OS.

In order to delineate the correlation of HIF-1α-miR-18b-5p–PHF2, we evaluated the expression of HIF-1α, miR-18b-5p and PHF2 in our clinical specimens. The results showed that expression of miR-18b-5p had a positive correlation with HIF-1α, while negative correlation with PHF2 (Fig. 6a,b). Meanwhile, expression of HIF-1α had a negative correlation with PHF2 (Fig. 6c). In summary, our results depicted that hypoxia driven HIF-1α enhanced the expression of miR-18b-5p in transcriptional level via binding to the HRE site of pre-miR-18b-5p promoter. The upregulated miR-18b-5p decreased PHF2 expression in post-transcriptional level by binding to the 3′UTR of PHF2 mRNA, leading to the increased proliferation and metastasis of OS cells (Fig. 6d).

## Discussion

Rapid proliferation and pulmonary metastasis were validated as the main cause of poor outcomes in OS patients. Although great progress has been achieved in the options of treatment, the 5-year survival rate of OS is still low. Early and correct diagnosis is not only the key to treating the disease successfully, but also an important guarantee for improving the prognosis and survival rate of patients. Since thousands of functional miRNAs have been identified in cancer, these small molecules can be utilized as biomarkers to diagnose the diseases and prognosis. In our present study, we identified miR-18b-5p was upregulated in OS cell lines compared with normal bones via GEO datasets. The in vitro and vivo assays validated that enhanced miR-18b-5p expression contributed to the increased proliferation and metastasis of OS. Mechanically, miR-18b-5p repressed the expression of tumor suppressor gene-PHF2 in post- transcriptional level. Furthermore, the upregulation of miR-18b-5p under hypoxia is due to the transcriptional regulation by HIF-1α. In this study, our results have enriched the mechanisms by which miRNA plays a carcinogen-promoting role in OS.

Increasing evidence reported that dysregulated miRNA exerts a vital role in increasing the ability of proliferation and motility of cancer cells. Here, we identified abnormal expressed miR-18b-5p in OS cells compared to normal bones through screening the GEO dataset. Reports have elucidated the involvement of miR-18b-5p in several other types of cancers, including ovarian cancer^34^, colorectal cancer^35^, breast cancer^36^, hepatocellular carcinoma^37^, gallbladder cancer^38^. Among these studies, miR-18b-5p were mainly absorbed by lncRNA, and acted as a tumor suppressor or promotor. In our exploration, we identified that miR-18b-5p exerted its function as a tumor promotor. Survival analysis suggested that high miR-18b-5p level was associated with poor prognosis in OS patients. Therefore, miR-18b-5p is a promising therapeutic target for cancer treatment in OS. While, miR-18b-5p can act on multiple target genes and signaling pathways, and the interference of miR-18b-5p may have a huge impact on other signaling factors. Thus, the application of miR-18b-5p in clinical treatment still faces great challenges. However, there is no doubt that our results provides new evidence and cutting points for tumor therapy.

miR-18b-5p played opposite roles in different tumors, suggesting that tumor microenvironment may have a certain influence on the expression and activity of miR-18b-5p. In our studies, we clarified that miR-18b-5p was upregulated by hypoxia induced HIF-1α at transcriptional level in OS. Meanwhile, miR-18b-5p medicated the enhanced proliferation and metastasis of OS cells via targeting PHF2 at post- transcriptional level. HIF-1α is a major transcription factor involved in initiating a variety of cellular processes required to restore oxygen homeostasis by regulating the expression of various genes^39^. Some JmjC demethylases has been revealed to be direct targets of HIF transcription factors, suggesting that they are involved in the function of hypoxia response^40^. Meanwhile, the research reported that PHF2 was significantly increased in HepG2 cells under hypoxic conditions^40^. In our studies, we found that PHF2 was remarkably downregulated by HIF-1α-driven miR-18b-5p in OS cells under hypoxia. Of course, the regulation mechanism of PHF2 in OS under hypoxia still needs to be further explored and verified.

In summary, our results depicted that hypoxia driven HIF-1α enhanced the miR-18b-5p expression in transcriptional level via binding to the HRE site of miR-18b-5p promoter. The upregulated miR-18b-5p decreased PHF2 expression in post-transcriptional level by binding to the PHF2-mRNA -3′UTR, leading to the increased proliferation and metastasis of OS cells.

## Material and methods

### Human tissue specimens

The histologically conformed cancer tissues and adjacent cancerous tissues of 60 OS patients were collected from Huazhong University of Science and Technology Union Shenzhen Hospital between 2016 to 2018. All specimens were obtained during surgery and immediately frozen in liquid nitrogen in accordance with the agreement of the ethics committee. And 60 patients were enrolled in clinical follow-up data for 3 years. OS patients with miR-18b-5p expression above or below the 50th percentile were classified as high or low miR-18b-5p group. All experiments were approved by the medical ethics committee affiliated to Huazhong University of Science and Technology Union Shenzhen Hospital and were carried out in accordance with relevant guidelines and regulations. All patients signed informed consent before recruitment.

### Cell culture

Human OS cell lines (KHOS, U2OS, MG63, 143b, Saos-2, SJSA and MNNG) and normal human osteoblast (hFOB 1.19) were gotten from the Cell Bank of China Academy of Sciences (Shanghai, China). U2OS, MG63 and MNNG cells were cultured using α-MEM medium (HyClone, USA) supplemented with 10% FBS (Gibco, USA). Saos-2 cells were cultured in RPMI-1640 medium (Gibco, USA) supplemented with 15% FBS. hFOB1.19, 143b, KHOS and SJSA cells were cultured in DMEM medium (Thermo Fisher Scientific, Waltham, MA, USA) supplemented with 10% FBS. All the culture medium was added 100 U/mL penicillin and 100 mg/mL streptomycin. The human OS cell lines were cultured in a 37 °C humidified incubator with 5% CO2. hFOB 1.19 cell lines grew at 33.5 °C with 5% CO2. For the hypoxia culture condition, cells were cultured under 1% O2, 5% CO2, and 94% N2 in a hypoxia incubator chamber. Hypoxic incubation time is 24 h unless otherwise specified.

### Western blot

Cells (50–80 × 10^5^/per sample) were lysed using 160 ul RIPA protein lysis buffer (Biotime, Hangzhou, China), which was supplemented with protease inhibitors (Roche, Basel, Switzerland) on ice for 30 min. Then the lysate of cells were centrifuged at 12,000*g* for 20 min at 4 °C. BCA™ Protein Assay Kit (Pierce, USA) was used to quantify the protein concentration. Added an appropriate amount of 5 × SDS-PAGE protein loading buffer in the ratio of 4:1 to the protein lysates, and adjusted the concentration with PBS. Afterwards, the cell protein lysates (10 ul/per sample) were separated by 10% SDS-PAGE and transferred to PVDF membranes (Millipore, Danvers, MA, USA) according to the provided directions. Incubated the membrane in 5% non-fat milk by shaking on a shaker for 1 h. Subsequently, the primary antibodies against PHF2 (Abcam, ab124434) and HIF-1α (cell signaling technology, #36169) were incubated with membranes overnight at 4 °C. After washing with PBST three times, the membranes were incubated with secondary antibodies (anti-Rabbit or anti-Mouse) for 1 h. Finally, the membranes were washed with PBST and imaged using ECL Western Blotting Substrate. Bio-Rad ChemiDoc XRS system (Bio-Rad, CA, USA) was applied to collect the chemiluminescent signals and quantify the intensity of the bands. β-actin (Abcam, ab8226) served as loading control.

### MTT assay

MTT assay was conducted to assess the capacity of cell proliferation. Briefly, 2000 pre-treated cells were cultured in 96-well plate with 6 replicates per sample for 5 days. And three blank replicates were used as controls. At the same time of each day, 20 µL of 3–(4,5-dimethylthiazol-2-yl)-2,5-di-phenyltetrazolium bromide (MTT, Sigma, St. Louis, MO) was added into each well. After intubation at 37 °C incubator in the dark for 4 h, the medium was removed and 150 µL of DMSO (Sigma) was added into the wells to dissolve the formazan crystals. Then the absorbance was detected at 570 nm via a microplate reader (Thermo Fisher Scientific). The absorbance was obtained by subtracting the average absorbance of the three control replicates from the average absorbance of the samples. The experiments were repeated three times independently.

### Transwell assay

Transwell assay was performed to evaluate cell invasion and migration. Firstly, the treated cells (5 × 10^4^), suspended in 200 μl of serum-free medium, were added on Matrigel-coated and uncoated upper chambers for invasion assay and migration assay, respectively (BD Bioscience, USA). Culture medium containing 10% FBS was added to the lower wells. After incubated at 37 °C for 24 h, the cells were fixed using 4% paraformaldehyde and stained using crystal violet. Then the cells on the membrane of transwell chambers were imaged using a microscope (Olympus, Japan). The number of invaded and migrated cells was observed in 5 random fields.

### Transfection

The miR-18b-5p mimic, mimic negative control (NC), miR-18b-5p inhibitor, inhibitor NC, small interfering RNAs targeting HIF-1α and scrambled NC siRNA were brought from GenePharma (Shanghai, China). For stable transfection, miR-18b-5p was constructed into lentiviral vector (LV), with empty vector as a negative control. The LV-miR-18b-5p and LV-miR-NC were purchased from GeneChem (Shanghai, China) and transfected into MG-63 cells according to the given instruction. The transfection efficiency was shown in Table S1. The overexpression plasmid of PHF2 and scrambled vectors (Table S2) were purchased from GeneChem (Shanghai, China). Lipofectamine 2000 (Invitrogen) was utilized for the transfection. For the co-transfection of miR-18b-5p mimic and PHF2 overexpression plasmid, appropriate number of cells (20 × 10^4^/well) were inoculated in 6-well plates 1 day before transfection. And 4 μg PHF2 overexpression plasmids and 50 nM miR-18b-5p mimic were co-transfected into MG-63 and 143b cells. And 48 h later, the cells were harvested for further analysis. qRT-PCR and Western blotting was conducted to assess the transfection efficiencies. The corresponding sequences were listed in Table 1.

**Table 1:** Information of the siRNAs and miRNA mimic.

Name	Target sequence or siRNA sequence (5′-3′)
si-HIF1α	UUCAACUUUGGUGAAUAGCTT
NC-siRNA	Sense: UUCUCCGAACGUGUCACGUTT Antisense: ACGUGACACGUUCGGAGAATT
miR-18b-5p mimic	UAAGGUGCAUCUAGUGCAGUUAG
miR-18b-5p inhibitor	CUAACUGCACUAGAUGCACCUUA
Negative control mimic	UUGUACUACACAAAAGUACUG
Negative control inhibitor	CAGUACUUUUGUGUAGUACAA

### Reverse transcription quantitative polymerase chain reaction (RT-qPCR)

The cells (50–80 × 10^5^/per sample) and 100 mg cancer tissues/adjacent cancerous tissues were prepared for total RNA isolation. TRIzol reagent (Invitrogen, CA, USA) was utilized to isolate the total RNA from cells or tissues. The miRNA First Strand cDNA Synthesis Kit (Thermo Scientific, Waltham, MA, USA) and PrimeScriptTM RT reagent Kit (Takara, Dalian, China) were used to synthesize the cDNA (Applied Biosystems). The extracted RNA was quantified using a microspectrophotometer. Total RNA (1 µg) was used for miRNA first strand cDNA synthesis. Total RNA (500 ng) was used for the cDNA synthesize. The product of reverse transcription was diluted 30–50 times for qPCR. SYBR®Premix Ex TaqTM (TaKaRa) was used to conduct qPCR based on the given protocols. Endogenous U6 snRNA served as control to normalize the expression of miRNA. β-actin acted as control for the expression of mRNA. The primers for genes used in the study were listed in Table 2.

**Table 2:** The sequence of PCR primers.

Primer	Sequence
miR-18b-5p	Forward: 5′- TGTGCAAATCCATGCAAAACTGA -3′ Reverse: 5′- GTGCAGGGTCCGAGGT -3′
U6	Forward: 5′- CTCGCTTCGGCAGCACA -3′ Reverse: 5′- AACGCTTCACGAATTTGCGT -3′
PHF2	Forward: 5′- GCCTCTAACCACAGCGAGAT-3′ Reverse: 5′- GTAGATCCAGCCTGAGGGGA -3′
HIF-1α	Forward: 5′- AGTTCCGCAAGCCCTGAAAGC -3′ Reverse: 5′- GCAGTGGTAGTGGTGGCATTAGC -3′
β-actin	Forward: 5′- AGTCCTACGGAAAACGGCAG -3′ Reverse: 5′- CGGCTATTCTCGCAGCTCAC -3′
ChIP primer	Forward: 5′- TAATGCTCCCGTTGAAGT -3′ Reverse: 5′- TACCAGAGTGCGGTTGAG -3′

### Luciferase assay

The procedure was performed as previously described^41^. PGL3 vector (Table S2) wild and mutant reporter plasmids carrying the predicted binding sites of miR-18b-5p in PHF2 mRNA- 3′UTR sequence and mutant (PHF2-WT: Table S3, PHF2-MUT: Table S4) were transfected in MG-63/143b cells with miR-18b-5p overexpression or inhibition to identify the interaction between miR-18b-5p and 3′UTR of PHF2 mRNA. PGL3-basic plasmids containing the promoter sequence of miR-18b-5p (a 2-kb sequence upstream of miR-18b, Table S5) were transfected in MG-63 and 143b cells culture under normoxia or hypoxia to evaluate the transcriptional activity of miR-18b. The plasmid pRL-TK carrying the Renilla luciferase was co-transfected into the cells as internal reference. Lipofectamine 2000 (Invitrogen) was applied to conduct the transfection. 48 h later, the luciferase activity was quantified using a Dual-Luciferase Reporter Assay System (Promega, USA) based on the manufacturer’s instructions. Specifically, the 5 × passive lysis buffer (PLB) was provided in the system and diluted to 1 × PLB as the given instructions. 48 h after cells co-transfection, each well was cleaned with PBS and added 1 × PLB. Subsequently, the culture plate was placed in a shaker for 25 min to allow the cells to be completely lysed. The lysate was then transferred to a 96-well plate protected from light, and the pre-mixed Luciferase Assay Reagent II was added to each well for 2 s to detect the first luminescence value (firefly luciferase activity). After determination, pre-mixed Stop&Glo Reagent was added to each well for 2 s, and the second luminescence value was Renilla luciferase. Finally, the data was presented as the ratio of firefly luciferase activity to Renilla luciferase activity.

### ChIP assay

The ChIP assay was conducted using Chromatin Immunoprecipitation (ChIP) Assay Kit (Millipore, Catalog # 17-295) according to the provided instructions^42^. Briefly, 1 × 10^6^ MG-63 cells were cultured under normoxia or hypoxia on a 10 cm dish. And 24 h later, the cells were treated with 1% formaldehyde for DNA–protein crosslinking. After removing the formaldehyde and washing cells twice with protease inhibitors containing ice cold PBS, the cells were scraped and collected through centrifugation (4 min, 2000 rpm, 4 °C). The cells were lysed with 200 µLSDS Lysis Buffer for 10 min on ice. The DNA of cells was sheared by ultrasonic treatment to lengths between 200 and 1000 bp. The ultrasonically treated cell lysates were centrifuged at 4 °C at 12,000×*g* for 10 min. One percent of supernatant was kept as input. The rest of supernatant was divided into two parts and incubated with anti-HIF-1α (cell signaling technology, #36169) or rabbit-IgG (cell signaling technology, #2729) antibodies at 4 °C overnight, respectively. The DNA–protein complex was precipitated with Protein A Agarose for 1 h at 4 °C. Then the pellet agarose was collected via gentle centrifugation (1 min, 1000 rpm, 4 °C). The supernatant was removed and the sediment was washed. The DNA fragments were purified and extracted using phenol/chloroform after reversing the cross-linking overnight at 65 °C. Finally, the purified DNA was detected and analyzed by qPCR. The gel electrophoresis on 2% agarose gel was utilized to analyze the PCR products. The sequence of PCR primers are indicated in Table 2.

### Biotin miRNA pull-down assay

The method was the same as the literature reported^43^. The 4 × 10^6^ MG-63 and 143b cells were transfected with 50 μM single-stranded biotinylated miR-18b-5p or miR-NC, designed and synthesized by the GeneChem (Shanghai, China). And 36 h later, the transfected cells were harvested and lysed by the freeze–thaw lysis method. Freshly prepared cell lysate and the blocked streptavidin-coated magnetic beads were incubated on a bench-top nutating mixer at room temperature for 1 h. After that, the beads were treated and washed with freshly prepared ice-cold pull-down wash buffer on the magnetic separator. Finally, the beads were resuspend in 100 µL of nuclease-free water for further analysis. Extract the total RNA from the resuspended beads. Finally, the purified RNA was further analyzed by RT-qPCR analysis.

### In vivo assay

BALB/c nude mice (male, 4 weeks old) were brought from Shanghai Laboratory Animal Research Center (Shanghai, China). For the xenograft model, 16 mice (8 each group) were subcutaneously injected with MG-63 (100 μl, 2 × 10^6^) transfected with LV-miR-18b-5p or LV-miR-NC in the right hip. The size of the tumor was recorded every 3 days. And 33 days later, the mice were sacrificed and the tumors were harvested and weighted. The tumor volume was detected every 3 days based on the equation *V* = 0.5 × *L* (length) × *W*^2^ (width). For the metastasis model, 16 mice (8 each group) were injected with MG-63 (100 μl, 2 × 10^6^) transfected with LV-miR-18b-5p or LV-miR-NC from the tail vein. The survival time of the mice was recorded. Lung metastasis was quantified by H&E staining. The animal experiments were reviewed and approved by the Animal Care and Use Committee affiliated with Huazhong University of Science and Technology Union Shenzhen Hospital and were carried out in accordance with relevant guidelines and regulations in compliance with the ARRIVE guidelines.

### Statistical analysis

All results were analyzed and presented as mean ± SD using the SPSS 18.0 software and GraphPad Prism 6.0 (GraphPad Software, San Diego, CA, USA). Student’s t test or one-way ANOVA was utilized to evaluate the differences of group. The results were an independent randomized trial with 3 replicates. Kaplan–Meier analysis was applied to analyze the survival of OS patients. The log-rank test was applied to compare two Kaplan–Meier survival curves (Table S6). Pearson correlation analysis was performed to assess the correlations between the expression levels of the two genes. *P* < 0.05 was considered significant (*,*P* < 0.05; **, *P* < 0.01; ***, *P* < 0.001, NS, no significant).

## Supplementary Information

Supplementary Information.
