# Supplementary material for: HIF-1α-mediated augmentation of miRNA-18b-5p facilitates proliferation and metastasis in osteosarcoma through attenuation PHF2
Source: Sci Rep. 2022 Jun 21;12:10398. doi: 10.1038/s41598-022-13660-w (PMC9213540; doi:10.1038/s41598-022-13660-w)

**HIF-1α-mediated augmentation of miRNA-18b-5p facilitates proliferation and metastasis in osteosarcoma through attenuation PHF2**

Peng Luo^1, #^, Yan-dong Zhang^2, #^, Feng He^1^, Chang-jun Tong^1^, Kai Liu^3^, He Liu^1^, Shi-zhuang Zhu^1^, Jian-zhou Luo^1^, Bing Yuan^4, *^


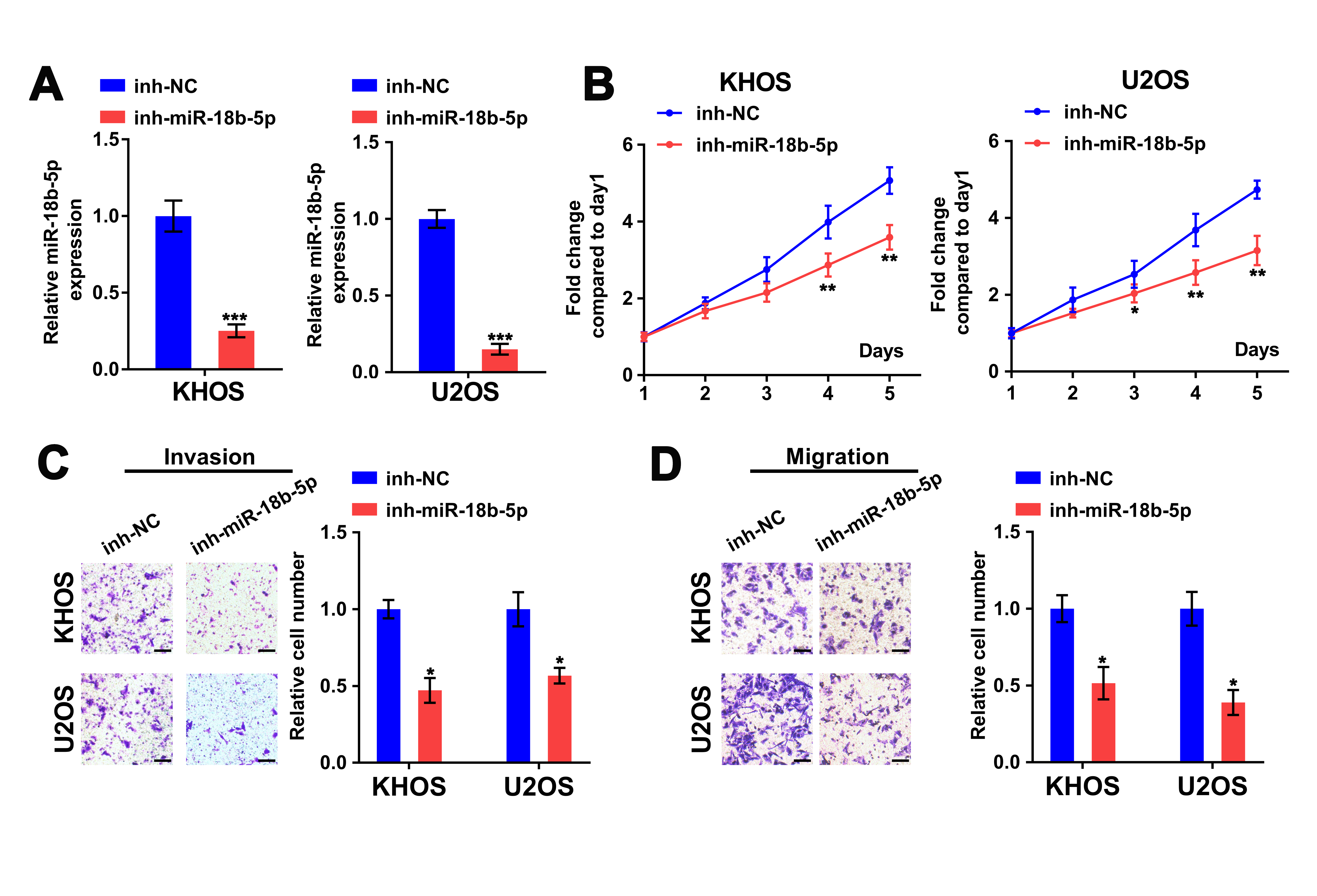
Figure S1: Downregulation of miR-18b-5p inhibits proliferation, invasion and migration of OS cells. (A) RT-qPCR was performed to detect the level of miR-18b-5p in KHOS and U2OS cells with miR-18b-5p inhibition. (B) The cell viability of KHOS and U2OS cells was evaluated by the MTT method. (C, D) The capacity of invasion and migration of KHOS and U2OS cells was valuated by the transwell assay. Scale bar: 100 μm.


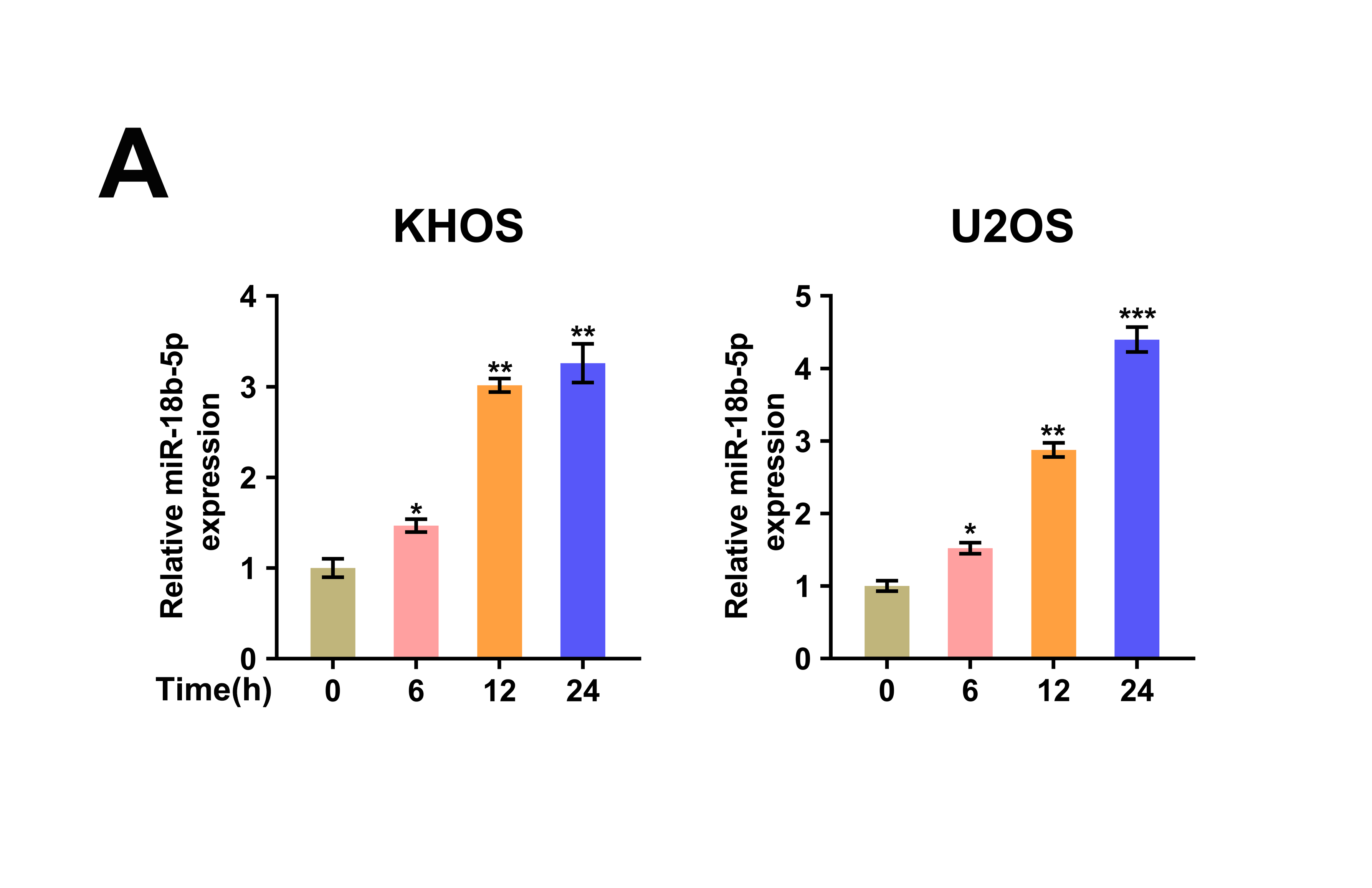


**Figure S2: Hypoxia induced the elevation of miR-18b-5p in OS. (A)** RT-qPCR assay showed miR-18b-5p expression in KHOS/U2OS cells cultured under hypoxia for 0h, 6h, 12h and 24h.

Table S1. The transfection efficiency of LV-miR-18b-5p


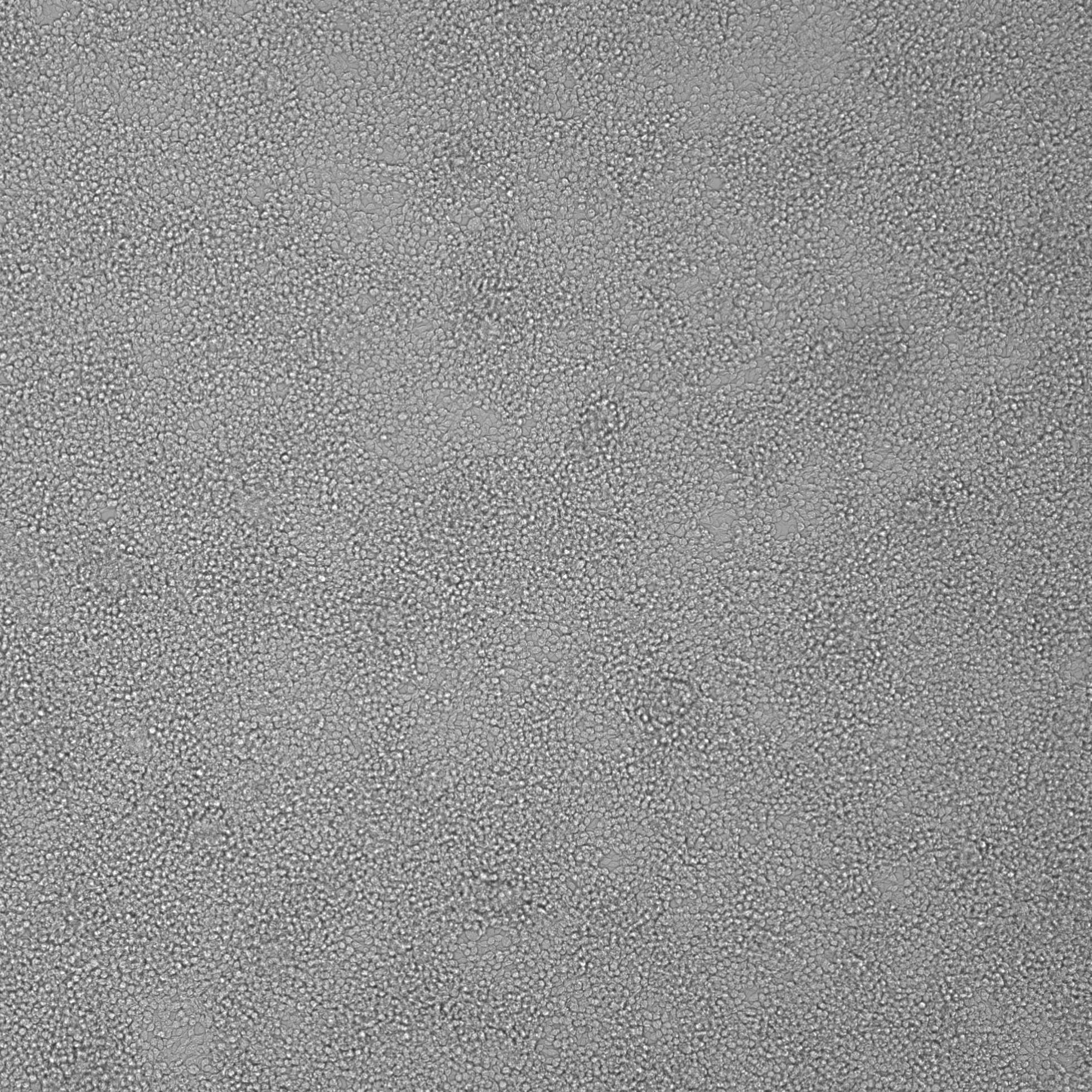

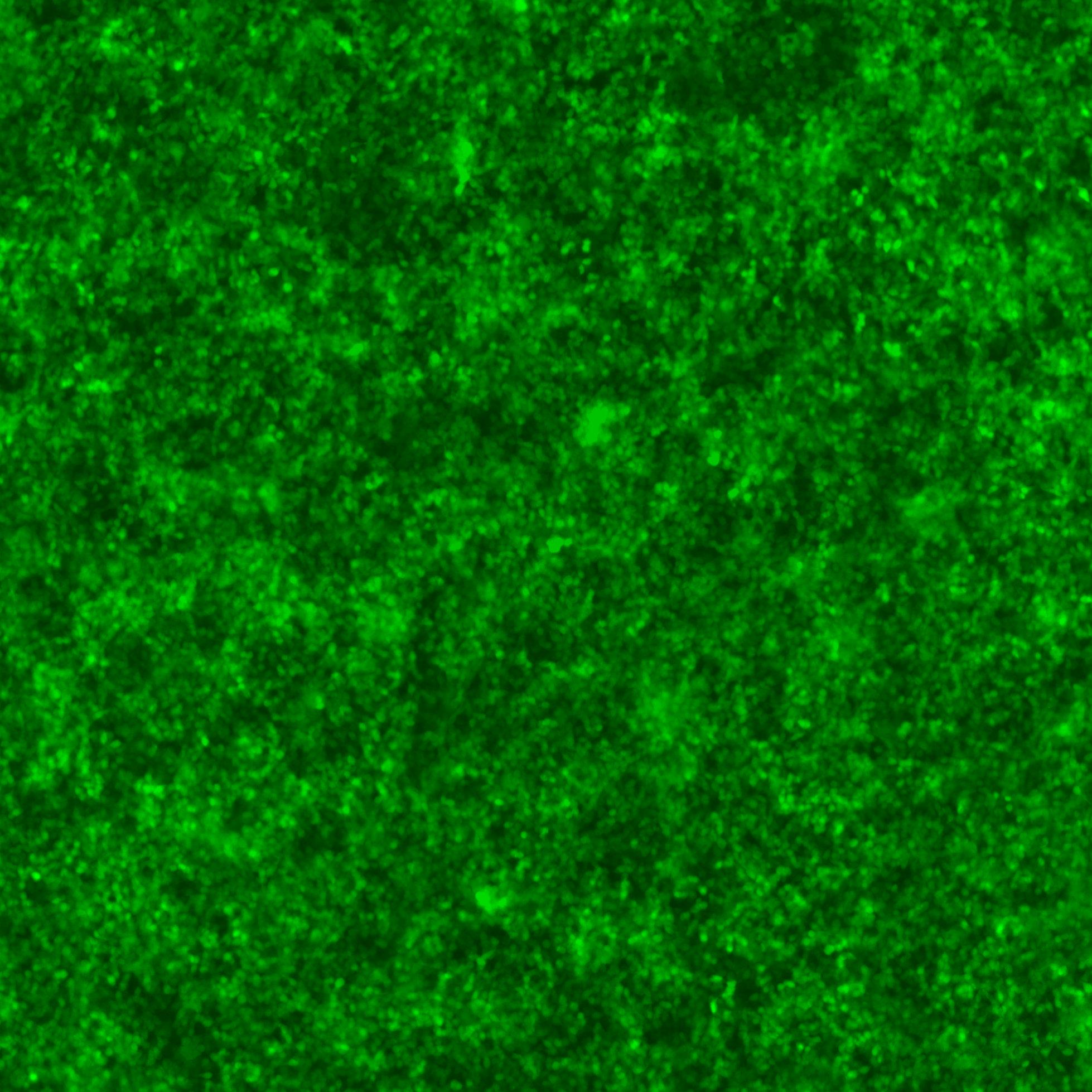


Table S2. The Vector structure of overexpression plasmid.

PHF2-OE

Carrier Name: GV230

The sequence of component: CMV-MCS-EGFP-SV40-Neomycin


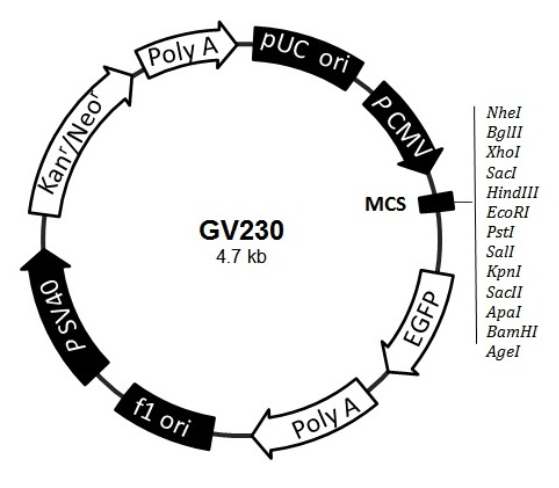


pGL3-control vector


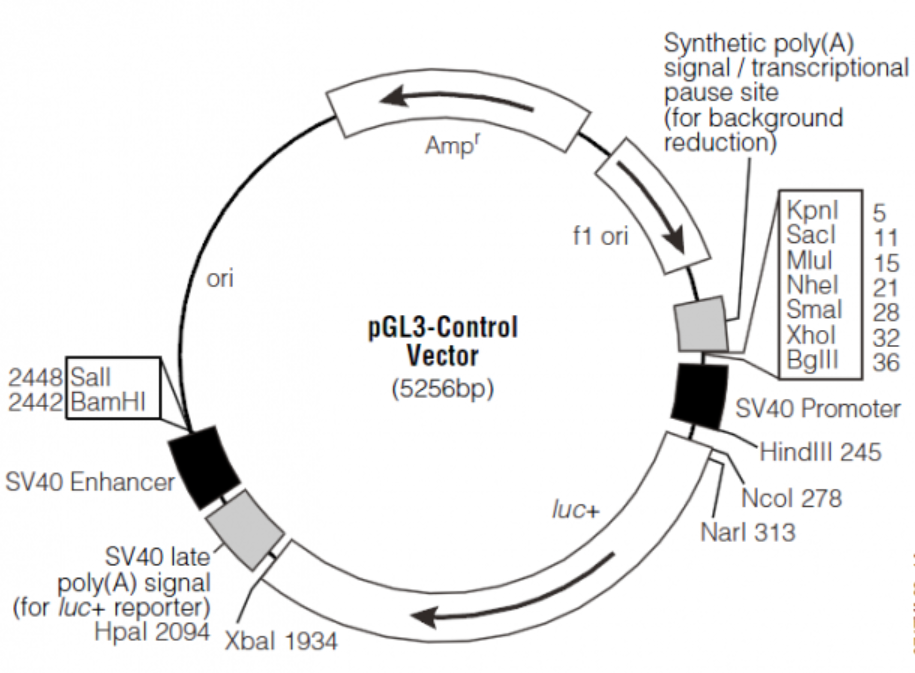


pRL-TK vector


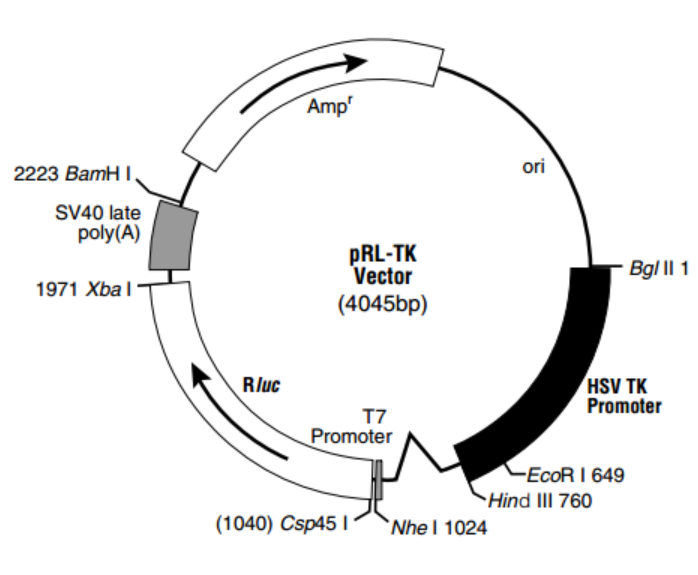


| Table S3. The sequence of PHF2 mRNA- 3’UTR sequence (Wild type) |
| --- |
| 1 gatttggaaa gccaggatcc ttctgctccg ctcaggaccc ccggagcccc gcgaaaacat  61 ctgcctccca ggagggtgcc gagctgcctc accagggagg gccttgcctc ttcccggctg  121 ccatctcccc aacaagcgtc tgtcccttca gccggcagag cgagcccagc gtggcccctc  181 aatttgaaaa tggacgtctt ttctcaagtt gctaagagtg atctgtccca gaaaagcggc  241 cctgcaagtt tgaggaccgc ttattccact ttaaggacag ccttcaggcc ccctgagcgt  301 gggtgtgatt gcagggcctc tgcagctctg ctgagagcat gagtccttca aggaagacag  361 agtgagccaa tgctcaccag ccccagagtc agagctggcc acaggctggc agcctccagg  421 ggcttaaaaa aaaaggcaaa gaacacagaa agaggaggag caagtgggat gtttatgtcc  481 ccccttctct tcctgagtga ttctcagcca agtccagaca gtgctcggcg ggtgaggaag  541 ggtctgcccc gagctttctg gttggcaggt ggcagcagga tggtgggtgt tcagcctgaa  601 tgcccaggag catttctggg gggcagctaa gactggcagc tgggttggtg tgttagcggg  661 caggggagcc attgtggggt ccccaggaaa gggcaagggc tcagccacat cttggggtct  721 gggaggccca ggctaagcca tgtggcaggg accgtcttgc cctgctggcc acactctgga  781 gaagcacttc tcagccaagg cacccctgcc ctgggactgg cagggcaggg gcaggggcag  841 ggacagtggc caggcggccc gaggacttac ggtcggcact tctctgttct cccgtgtcag  901 cgtgtggtgt cgcctgcatg ggtcgtacct ggatggtgtg tccaccatcg acacggaggg  961 gctggatttg tttctcaggc aatcctgtat tttaatttta gatgtatttc ctgaagcata  1021 tttttcatag aatgtagcgt gtaaatagct ttttaaataa cttctttttt ataagagtaa  1081 aagtatcttt aggaatttct ttctatagag ttcttcatta acatttatac gagttttttg  1141 ctgagtcaga tggacagttg ggttctgatg ctttttcctt ctcctttcct tttattatta  1201 ttattttttt cttttaagaa ctaaggtatt gcctgaaaaa caagtgatgt ctgtgcagcc  1261 ttacactctg tctttacaga agcaaatagt acacaaaaga tctatttcag acacattttg  1321 aagatgaatc ttcaacttta ataccagctc tttgttttcc ttgtatgatg aggggattgg  1381 gggatacagt tatttt**acta gcacctt**gtg aagtgtttcc gtgttttgtg atgctgtaat  1441 ttattaatgt ttgtagcttt ttatatttgt acatttctta tgagctttgt ttatataccc  1501 attacctgga tgtttttgtc cactgggaga ggcagcttgg tggaggcctt atccactccc  1561 acttgtcctg tttggaggga cgcagtccct agggcccgag actgggtggg agagggggag  1621 tctcacgggg ccccaggctt attcagaact ggtgttttta aagtttcctt taccctgccc  1681 ttgttgaaca tttatataat ctaacctgga catcaagctg ttctctctct ctcttttttt  1741 taattttatt attattattt tggcaacatg tacatttcta acaaagttta tcgtggctat  1801 taaagtgttt tatttcccaa ttcatattac tcttgtatcg agtccatgag gtctaaggca  1861 acttagatca aagttttaaa aaagtaaaaa tatttcaggt tttgtacaga a |

Table S4. The sequence of PHF2 mRNA- 3’UTR sequence (Mutant type)

| 1 gatttggaaa gccaggatcc ttctgctccg ctcaggaccc ccggagcccc gcgaaaacat  61 ctgcctccca ggagggtgcc gagctgcctc accagggagg gccttgcctc ttcccggctg  121 ccatctcccc aacaagcgtc tgtcccttca gccggcagag cgagcccagc gtggcccctc  181 aatttgaaaa tggacgtctt ttctcaagtt gctaagagtg atctgtccca gaaaagcggc  241 cctgcaagtt tgaggaccgc ttattccact ttaaggacag ccttcaggcc ccctgagcgt  301 gggtgtgatt gcagggcctc tgcagctctg ctgagagcat gagtccttca aggaagacag  361 agtgagccaa tgctcaccag ccccagagtc agagctggcc acaggctggc agcctccagg  421 ggcttaaaaa aaaaggcaaa gaacacagaa agaggaggag caagtgggat gtttatgtcc  481 ccccttctct tcctgagtga ttctcagcca agtccagaca gtgctcggcg ggtgaggaag  541 ggtctgcccc gagctttctg gttggcaggt ggcagcagga tggtgggtgt tcagcctgaa  601 tgcccaggag catttctggg gggcagctaa gactggcagc tgggttggtg tgttagcggg  661 caggggagcc attgtggggt ccccaggaaa gggcaagggc tcagccacat cttggggtct  721 gggaggccca ggctaagcca tgtggcaggg accgtcttgc cctgctggcc acactctgga  781 gaagcacttc tcagccaagg cacccctgcc ctgggactgg cagggcaggg gcaggggcag  841 ggacagtggc caggcggccc gaggacttac ggtcggcact tctctgttct cccgtgtcag  901 cgtgtggtgt cgcctgcatg ggtcgtacct ggatggtgtg tccaccatcg acacggaggg  961 gctggatttg tttctcaggc aatcctgtat tttaatttta gatgtatttc ctgaagcata  1021 tttttcatag aatgtagcgt gtaaatagct ttttaaataa cttctttttt ataagagtaa  1081 aagtatcttt aggaatttct ttctatagag ttcttcatta acatttatac gagttttttg  1141 ctgagtcaga tggacagttg ggttctgatg ctttttcctt ctcctttcct tttattatta  1201 ttattttttt cttttaagaa ctaaggtatt gcctgaaaaa caagtgatgt ctgtgcagcc  1261 ttacactctg tctttacaga agcaaatagt acacaaaaga tctatttcag acacattttg  1321 aagatgaatc ttcaacttta ataccagctc tttgttttcc ttgtatgatg aggggattgg  1381 gggatacagt tatttt**tgat cgtggaa**gtg aagtgtttcc gtgttttgtg atgctgtaat  1441 ttattaatgt ttgtagcttt ttatatttgt acatttctta tgagctttgt ttatataccc  1501 attacctgga tgtttttgtc cactgggaga ggcagcttgg tggaggcctt atccactccc  1561 acttgtcctg tttggaggga cgcagtccct agggcccgag actgggtggg agagggggag  1621 tctcacgggg ccccaggctt attcagaact ggtgttttta aagtttcctt taccctgccc  1681 ttgttgaaca tttatataat ctaacctgga catcaagctg ttctctctct ctcttttttt  1741 taattttatt attattattt tggcaacatg tacatttcta acaaagttta tcgtggctat  1801 taaagtgttt tatttcccaa ttcatattac tcttgtatcg agtccatgag gtctaaggca  1861 acttagatca aagttttaaa aaagtaaaaa tatttcaggt tttgtacaga a |
| --- |

Table S5. The promoter sequence of miR-18b-5p (a 2-kb sequence upstream of miR-18b)

>5' Flanking sequence chromosome:GRCh37:X:133304147:133306146:1

CACATTATAAGAAGCAGCTCAAAAGCATCAACTACTTAATTATGCACAAACTACAGTTCT

CAGTAGCCATTGACTAAATCACCATGGTAATGTAAGAAGTGCTTACATTGCAGTAGATCT

CAAAAAGCTACCTGCACTGTAAGCACTTTTACATGGCCAAGGCCTATTCCTGTAGCAAAA

TTTTAAGTCTTCCAGGAGCTCTTCTTTTCCGTCCTCACAGATCCATGCAGACTCACCTGG

TTAAAACAACAAAACAAACACACACATATTAGAAACTAAACAAGTAAACTACTAGTTAAT

ATTCCTGAAACAGGCTTTCCTTTGGCTTACAGAGTCTAAGCTAGGTTTTGTGGTTTCAAC

CAAATCCTGAGAAATATTAATAATCTCAATTATTTACACAGAATTGAACACGAATAACAC

TGGCAACTGCAAAAAGTCCAATTCTTATGTATAAGCAGATTTTGGACTCCCCCTCTCCAT

CTAAGAAAATTTGATATCTGCATCTCAGTAATTTGCAGGCACATTCTATCCACCCTGATT

TTATAAGAGGAGGACCTAGAGAAAGGAGCATGTAAAACCCAGGATGAGTATTCGAGTCTT

CAGATCATTCAGAGCTTTTGGGCTCCATACGGAAAGTGACTGTTAGATTAAATGAATATT

CCCAGCCCCCACCCCATTAATAGAAAATGAGAGAAATTTTAGAGCTGGTTTAAGAAACAC

AGCCTTACAGTCTTCACATAATGCACAGATGGCTGAGAGATGTCACATTTTGATCTATTT

CAAAGGGCTCTACTTCTGTTTAAACATTTATGGACCCATTTTCTCCATTAAACCAAAGTA

GGCAGATTAGCTCTTCTTCCCTTCATGTCATTATCTTCCATAAGAAAACCTTCTCCCATT

TCCAAGTGCTTATTTTACCTAAGAACATACAGCAAGGAAGTGAGCCTCTATGTGAAAAAG

GCTCACAGGGTCCCCTATCTCTTCCATGATGGGGTAACTTTTGTTTAAAAGAAAAACAAA

AACGTTTGGGACAGAATCTGTAATACGTTAAACGAGTGAGGGAATTTTAAATGTTGAACT

TTCGTCACGATATCTTATGCCACTAAATGAGTCTTTAGAAGTCTTTACAAACTAAACTGA

TTTATAAAACTTATACATCAATCTGGAATCAATTTTCTATTAGGGAAGAATGGATTTAGT

ATAGAAATGAGAAAAATAACATTTTTCCCCTTAAATTGGGTTTCTACACCTGTGAAGATT

TCTTTTCCATTAAAAAAAAAATACTCCAAACTTGACTCAAAATGCATAGTATTTGCCAAC

CCAACCTGGCCAAACTGAGCCCCACAGCCATCAGACAGGGCGCCCTTCTAGTTACTACCT

TTTGCTAATTAGCGGTTCATCTGGCATCCCTCGCGCACGCTGGGCAGTCACTCTCCAATC

CCCTTAAATTGCAAATAATAATAATAATAATAAGGAAAAGAAAGAAAGAAGTCCTTCCCT

GTACGCCTTTGTGGCTCCCTTTTGCCTCTTCCCGCGTCTCTCACACCCTTACCTAATGCT

CCCGTTGAAGTCCGCGAGCAGCCCGCGCGTCCAGGGTGTGCTCCAGCTGCTCACACCACG

CGCTCCAGTCCTCGTGCAGCCTCAGCCGCCGCCGAAATTAAAGAGAAAGAAACAAAGCAT

CATGGGAAACGGAGTTCTCCATTCAAAGCACCAAAAACGCGCCAAGCCCACGCCTCCCTC

CCCCTCCATTCCCGGGTTGGTGGGATAGAAGCCAAGAGGGAGGGGAGTCCCTTTTTTCCC

TCC**ACGTG**CACCCCCTCATTTCACACCCTCACCACCAAAGTTTTCCAGGAACCTAAAGGT

CAACGGTGGAGAAGAAAAGGGGAGTCGTTTCCCGCGCGCTGCCCGCTACCCCTCAACCGC

ACTCTGGTACTTTGCTTTTCTCAAGTCCCGCCCGCTCCGTCCCCTCCCACCCAACCAGTC

AACTCTACCGGATCACAGCT

Table S6. The survival time of OS patient

| Survival time (Months) | miR-18b-5p-high | miR-18b-5p-low |
| --- | --- | --- |
| 3 | 1 | 0 |
| 8 | 1 | 0 |
| 10 | 2 | 1 |
| 13 | 2 | 0 |
| 14 | 2 | 0 |
| 15 | 1 | 0 |
| 18 | 0 | 1 |
| 20 | 1 | 0 |
| 21 | 0 | 1 |
| 23 | 0 | 1 |
| 24 | 1 | 0 |
| 33 | 0 | 3 |
| 38 | 1 | 0 |
| 40 | 2 | 0 |
| 55 | 1 | 0 |
| 60 | 15 | 23 |

Unprocessed original images


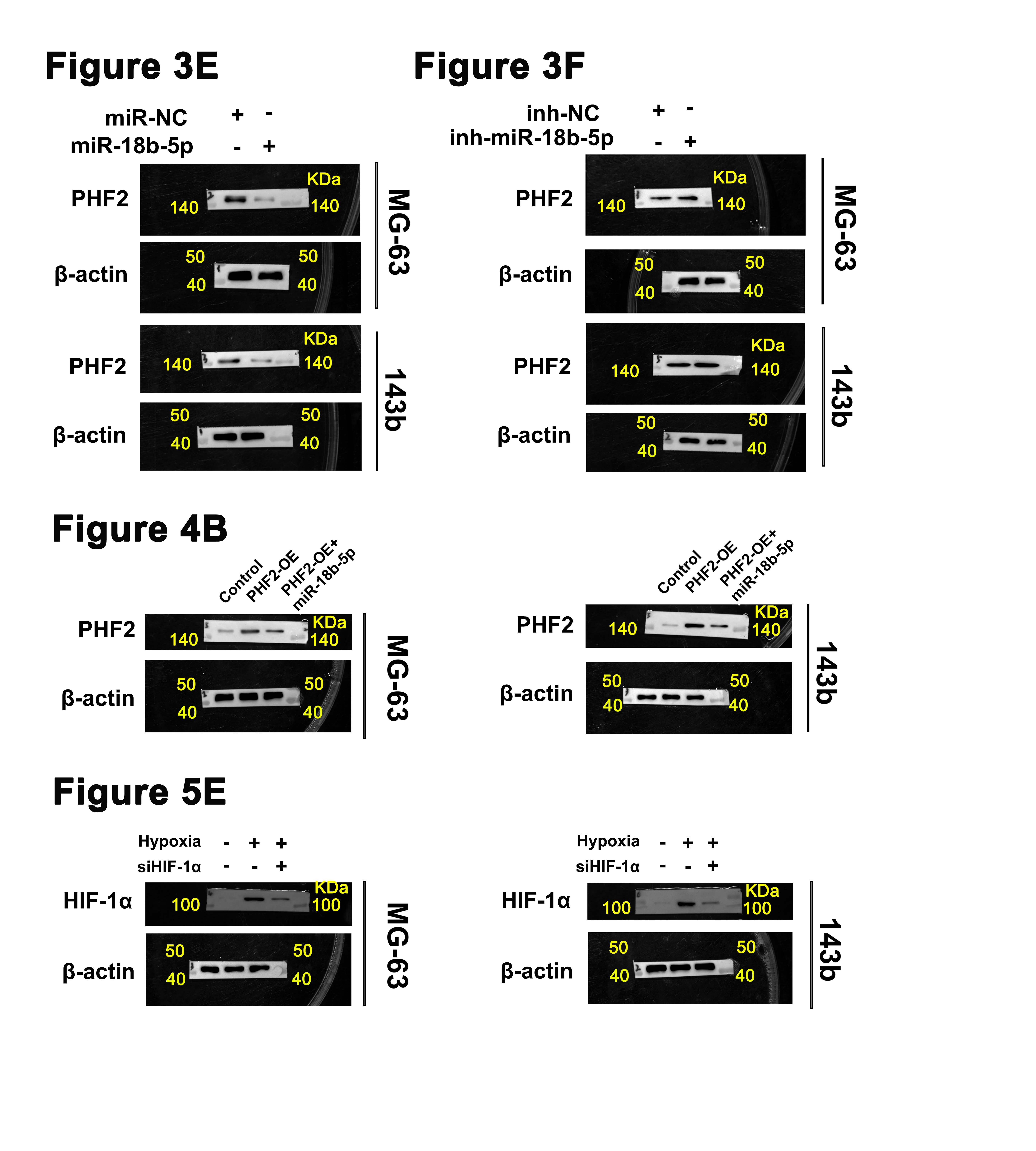


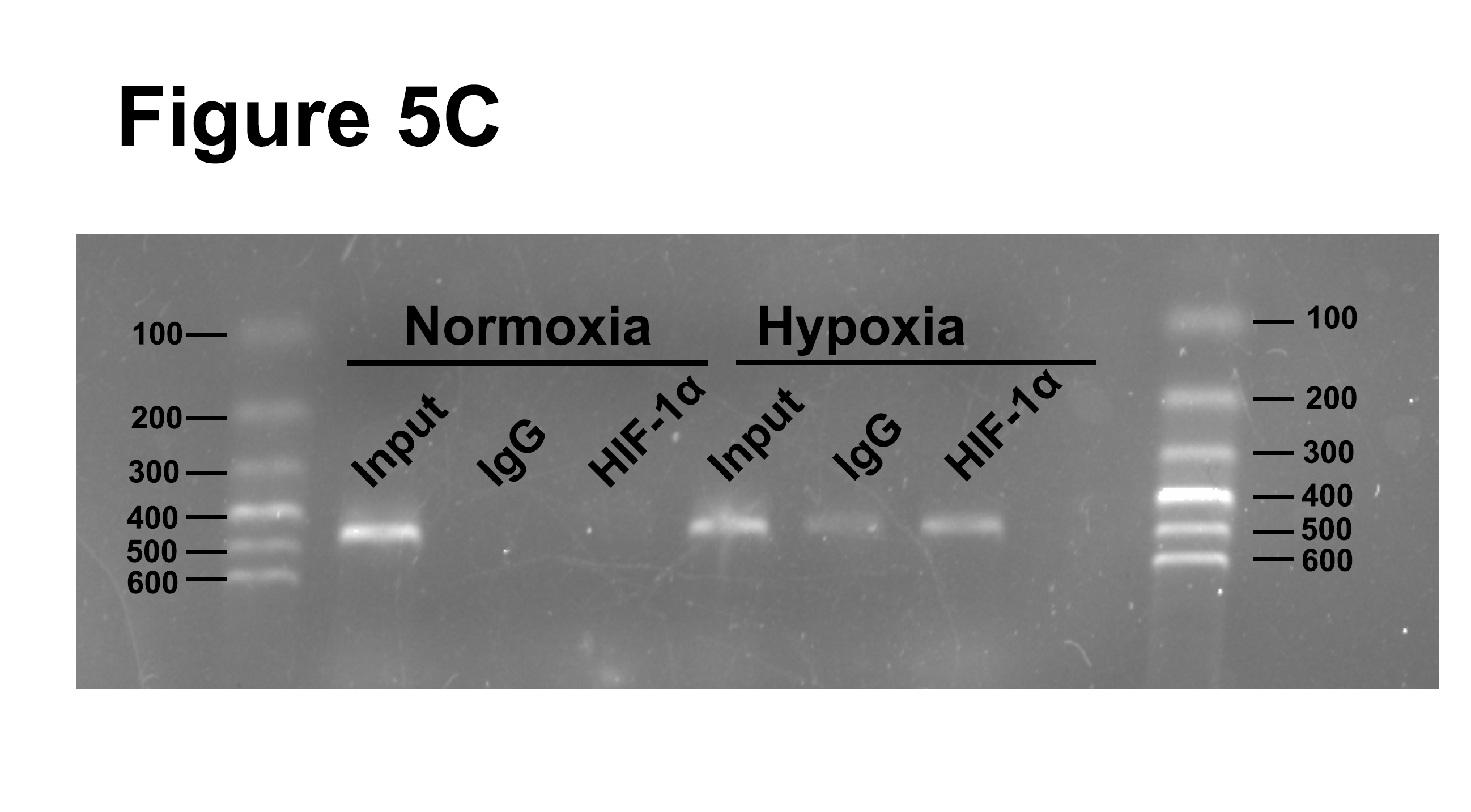

Supplement: Supplementary file 1 — Supplementary Information. [file 41598_2022_13660_MOESM1_ESM.doc]
